# Electrochemical Detection and Capillary Electrophoresis: Comparative Studies for Alkaline Phosphatase (ALP) Release from Living Cells

**DOI:** 10.3390/bios10080095

**Published:** 2020-08-11

**Authors:** Thanih Balbaied, Anna Hogan, Eric Moore

**Affiliations:** Sensing & Separation Group, School of Chemistry and Life Science Interface, University College Cork, Tyndall National Institute, T12R5CP Cork, Ireland; thanih.balbaied@gmail.com (T.B.); annamaria.hogan@ucc.ie (A.H.)

**Keywords:** electrochemistry, linear sweep voltammetry, alkaline phosphatase, capillary electrophoresis, colorimetry, cancer cells, embryonic cells

## Abstract

Alkaline phosphatase (ALP) is one of the main biomarkers that is clinically detected in bone and liver disorders using optical assays. The electrochemical principle is important because point-of-care testing is increasing dramatically and absorbance techniques hardly compete with the medical revolution that is occurring. The detection of ALP using electrochemical detection is contributing to the integration systems field, and hence enhancing the detection of biological targets for pharmaceutical research and design systems. Moreover, in vitro electrochemical measurements use cost effective materials and simple techniques. Graphite screen-printed electrodes and linear sweep voltammetry were used to optimize the electrochemistry of the enzymatic product p-aminophenol using the enzyme kinetic assay. ALP release from embryonic and cancer cells was determined from adhesion cell culture. Additionally, capillary electrophoresis and colorimetric methods were applied for comparison assays. The resulting assays showed a dynamic range of ALP ranging from 1.5 to 1500 U/L, and limit of detection of 0.043 U/L. This was achieved by using 70 μL of the sample and an incubation time of 10 min at an optimal substrate concentration of 9.6 mM of p-aminophenol phosphate. A significant difference (*p* < 0.05) was measured between the absorbance assays. This paper demonstrates the advantages of the electrochemical assay for ALP release from cells, which is in line with recent trends in gene expression systems using microelectrode array technologies and devices for monitoring electrophysiological activity.

## 1. Introduction

ALP is a homodimeric enzyme. It has cofactors that include two zinc atoms and one magnesium atom in each subunit, which are important for the active sites [1]. ALP is found in almost all living tissue and can be expressed in four isoforms. Abnormal levels of ALP release can be seen in illnesses (such as liver disease, bone disorders, etc.), or during pregnancy or the rapid growth phases of childhood [2,3]. Recently, ALP has been identified as a potential cancer biomarker, and its early detection could potentially help in the treatment of the disease [4].

Electrochemistry allows integration in small devices, which is required for point-of-care testing. Electrochemical detection techniques are relatively easy to use and offer relatively fast detection. This is reflected in Clark’s work (1954) [5]. Electrochemical biosensors for ALP have rapidly developed since 1991, when Thomson and his team published their work comparing ALP resolution using an electrochemical assay to results from optical detection [6]. The principle was based on hydrolyzing o-phosphate by ALP in the presence of the p-aminophenyl phosphate (pAPP), where the non-electroactive substrate under the alkaline environment served to generate an electroactive substrate (pAP). This research has contributed to the development of ALP electrochemical detection [7,8,9,10,11].

Investigation of ALP release as a quantitative indicator of gene reporting in mammalian cells began in 1988 [12]. However, it was not until the development of the electrochemical immunoassay [13] that the electrochemistry of ALP release as a secretion enzyme from cells began to grow in popularity [14,15,16]. Almost ten years later, a proper protocol of ALP release was investigated using methods of molecular biology, biochemistry, enzymology, and chemiluminescence. These findings enhanced the detection of ALP using electrochemistry [17,18,19,20]. Kelso et al., 2000 exploited the electrochemical immunoassay, and were likely the first to detect secreted ALP in media using screen-printed electrodes and 2-naphthyl phosphate [21]. The initial protocols of ALP release in vitro used the same strategies used for β-galactosidase [22,23,24,25,26]. This was carried out by way of electrochemical analysis and using different samples, including single-cell analysis [27,28,29], biopsies [30], tissues [31] and accumulation of cells [32,33]. These strategies have their own disadvantages. For example, using a reducing agent (e.g., β-mercaptoethanol) caused inactivation of intracellular ALP [34,35,36]. Moreover, issues relating to electrochemical analysis, including buffer components, volusme of the electrolyte, and the parameters required during the assay, were not optimised [37,38,39]. This caused less concentration of ALP release.

**Figure 1:**
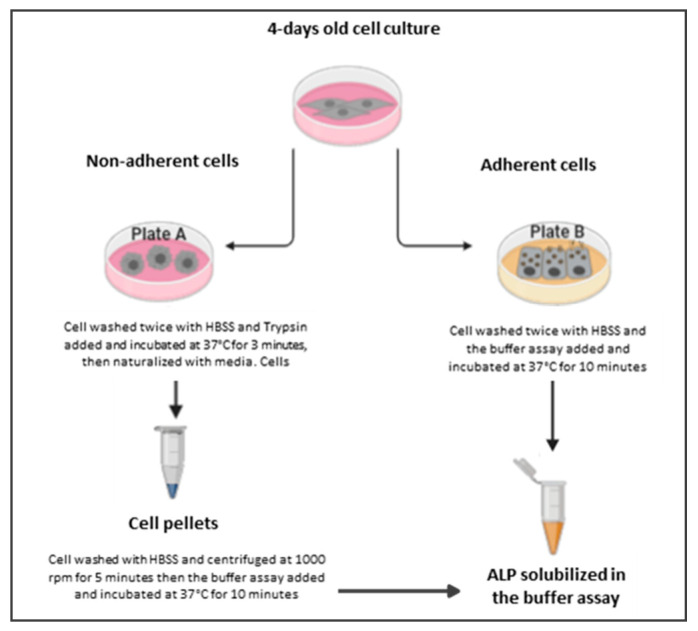
A schematic diagram of ALP release. Plate A describes the methods of determination of ALP release from detached cells, where cells are washed with Hank’s balanced salt solution (HBSS) and undergo trypsinization, washing and centrifugation before being exposed to the buffer assay. Plate B describes the methods of determination of ALP release from attached cells, where cells are only washed before being directly exposed to the buffer assay.

So far, investigations of ALP release from MCF-7 breast cancer cells, A549 lung cancer cells, and HT-29 colon cancer cells have only been carried out using fluorescence [40]. The optimization achieved as a result of spectroscopy would be negatively affected by the limitations (e.g., the cost, and the size of instruments). Accordingly, this paper will explore the parameters of the enzyme assay and apply the electrochemical assay for ALP release from living cells. Therefore, linear sweep voltammetry was used because it has a wide potential window, as it is a droplet-based assay and requires no stirring solution. These are important factors for point-of care application. The incubation time of the sample, the kinetic enzyme assay of the substrate, and calibration plots were studied in order to carry out regression analysis using the two methods illustrated in plate A and plate B (Figure 1). Separation techniques that included capillary electrophoresis were also used. Capillary electrophoresis was used because it distinguishes isoenzymes alongside the standard methods of colorimetry [41]. Finally, the assays were compared in order to assess the sensitivity of electrochemistry analysis.

## 2. Methodology

### 2.1. Chemicals and Instruments

Alkaline phosphatase (ALP, calf intestinal phosphatase) or 4-aminophenol (p-AP) was purchased from Sigma (Ireland) and used to make stock solution at a final concentration of 1500 U/L or 5 mM. The alkaline buffer assay at pH 9.5 was made by adding sterile deionized water at grade 18 MΩ provided from Tyndall National Institute (UCC), which contained diethanolamine (DEA) 1 M, magnesium chloride (MgCl_2_) 8 mM, sodium chloride (NaCl) 50 mM, potassium chloride (KCl) 100 mM, para-aminophenol phosphate (p-APP) and HCl purchased from Sigma (Wicklow, Ireland). The electrochemical measurements were performed on screen printed carbon electrodes with each individual sensor consisting of a graphite working and counter electrode and Ag/AgCl reference electrode (graphite-SPE) (Kanichi Research Limited, England, UK) with a working volume of 70 μL performed on palmSens portable poteniostat (Palm Instruments BV, Houten, The Netherlands). The graphite-SPE was cleaned before using for 20 min by Plasma Surface Treatment (Harrick plasma, Ithaca, NY, USA). Separations were performed using 50 μm i.d. and 375 μm o.d. fused-silica capillary (CM Scientific Ltd., Silsden, UK). Agilent 7100 Capillary Electrophoresis System (Waldbronn, Germany) the software Agilent Chemstation (Version B.02.01) were also used.

### 2.2. ALP Release and Cell Culture

In order to release ALP from cells, the following steps were performed according to the protocols developed by Thanih et al. [42]. The first step was to enhance ALP release in 4-day-old culture by seeding cells as a monolayer on 48-well plates (Sigma, Ireland), incubating at 37 °C and 5% CO_2_ (incusafe Panasonic incubator) and changing media every two days. The cell lines used during this study were purchased from ATCC (UK), including mouse embryo fibroblast cells (Balb/c 3T3 Line), breast carcinoma epithelial cells (MCF-7 Line), lung carcinoma epithelial cells (A-549 Line), and colon carcinoma epithelial cells (Ht-29 Line). The media, supplements, and washing buffer used for culturing cells were Dulbecco’s modified Eagle’s medium (DMEM), minimum essential medium Eagle (MEME) and McCoy’s 5A medium, newborn calf serum (NBCS), fetal bovine serum (FBS), and Hank’s balanced salt solution (HBSS), which were purchased from Sigma (Ireland). Table 1 summarises the cell numbers used and the composition of media. The cells were sub-cultured three times before seeding began under aseptic conditions using a cell culture hood (Esco Airstream^®^ Class II).

### 2.3. Stabilization of Graphite Screen-Printed Electrodes

Graphite screen-printed electrodes (graphite-SPE) were first cleaned using iso-propanol and a plasmon cleaner device for 10 min or for 20 min and compared to non-cleaned electrodes. Potassium [Fe(CN)_6_]^3−/4−^ (1 mM) was dissolved in 1.0 M of KCl. [Fe(CN)_6_]^3−/4−^ solution (5 mL) was used to immerse the graphite-SPE. A Palmsens potentiostat device was used to carry out the cyclic voltammetry assay and to compare the data before and after the cleaning process. The cyclic voltammetry detection techniques were applied at scan rates from 5 mV/S to 200 mV/S and at initial potential of −0.2 V and final potential of 0.6 V vs. the Ag/AgCl reference electrode. The potential range of each scan rate of cyclic voltammograms was plotted against the response current. The reduction peaks’ current (ipc) and oxidation peaks’ current (ipa) were plotted versus the square root of the scan rate ((mV/S)^1/2^).

### 2.4. Optimization of Electrochemical Measurement

Samples of cells were freshly prepared at a concentration of 250 × 10^3^ cells/mL. The cells were washed twice with HBSS, centrifuged for 5 min at 1000 rpm and resuspended in the assay buffer. Graphite screen-printed electrodes were first cleaned using plasma cleaner for 20 min and linear sweep voltammetry measurements were carried out at potentials ranging from −1.2 V to 1.5 V vs. Ag/AgCl. The measurements were completed in triplicate at a volume of 70 μL and a scan rate of 100 mV/S. To determine a probable working range for the product pAP, serial dilutions of pAP, starting with 5 mM were prepared in the presence of 30 μL of sample mixed with 100 μM of pAPP. The linear sweep voltammetry was measured for each concentration at three separate electrodes. A free-pAP buffer was used as blank. A graph charting current response to concentration of pAP was prepared for regression analysis. The incubation time evaluation for ALP release was performed using 70 μL of a sample with 20 U/L of ALP activity mixed with 30 μL of pAPP 5 mM in buffer DEA and assessed at 5-min intervals over 60 min. NaOH (30 μL of 1 M) was added in order to stop the reaction. The reaction time was fixed to 10 min, and the activity of the sample was assessed with different concentrations of substrate range (5, 2.5, 1.25, 0.63, 0.31, 0.16, 0.08, and 0.04 mM pAPP). The optimal concentration of substrate obtained was used in a calibration plot of ALP release. The sample was spiked with various concentrations of ALP (calf intestinal phosphatase) at a range of 1–1500 U/L.

### 2.5. Linearity Performance of ALP Release vs. Cell Number

In order to complete a linearity trend of ALP release versus current density, different concentrations of cells were used (250, 125, 63, 31, 16, 8, and 3 × 10^3^ cells/mL). The cells were incubated in the assay buffer for 10 min before measurement. Linear sweep voltammetry was applied in triplicate in three separate graphite-SPEs at a final volume of 70 μL, against a free-pAPP buffer that was used as a blank. We measured it at 100 mV/S and a potential range from −1.2 V to 1.5 V versus the reference of Ag/Ag/Cl. A graph relating the concentration of cells to the current density was prepared for regression analysis.

### 2.6. Concentration of the Substrate pAPP from Adhesion Cells

Adherent cells have different responses from non-adherent cells in terms of releasing ALP. Therefore, the concentration of substrate pAPP needed to be assessed in order to allow for real-time monitoring of ALP. The serial dilution of pAPP, starting with 5 mM, was carried out in the range of 5, 2.5, 1.25, 0.60, 0.30, 0.16, 0.08 and 0.04 mM in the assay buffer. The range of diluted pAPP was incubated with cells for 10 min. The electrochemical assay was applied with a final volume of 70 μL against a free-pAPP buffer as blank. A graph relating the concentration of pAPP to current density fit by non-linear regression analysis to the Michaelis-Menten model for each cell line was completed.

### 2.7. Comparative Study of ALP Activity

The conditions of samples in the comparative methods are the same as those in the electrochemical method. Capillary electrophoresis or colorimetric measurements were carried out in triplicate at a volume of 70 μL at a range of the product pNP (15–500 μM) using the wavelength 405 nm. The serial dilutions of pNP were prepared in the presence of 30 μL of sample mixed with 6 mM of pNPP. A graph relating peak area of absorbance to concentration of pNP was prepared for regression analysis. The slopes and intercepts of the reaction were used for normalizing the data in molarity. The separation of samples was performed in a 50 μm inner diameter fused-silica capillary with a length of 15 cm. The data were collected and processed using the capillary electrophoresis system and Agilent ChemStation software. The running conditions included a voltage of 15 kV, a capillary temperature of 20 °C and a wavelength of 405 nm, and injection of the sample was for 5 s and 15 mbar. A calibration plot of absorbance assay of ALP activity was obtained using the same sample preparation in electrochemical measurement. ALP release was performed using 70 μL of a sample spiked with various concentrations of ALP (calf intestinal phosphatase) at a range of 1–1500 U/L dissolved in 30 μL of pNPP 6 mM in buffer DEA and incubated for 30 min. NaOH (30 μL of 1 M) was added to stop the reaction. The Michaelis-Menten model was used to fit the data. The resulting sole and intercept were determined using Vmax and km constants to normalize the data.

## 3. Results and Discussion

### 3.1. Stabilization of Graphite Screen-Printed Electrodes

**Figure 2:**
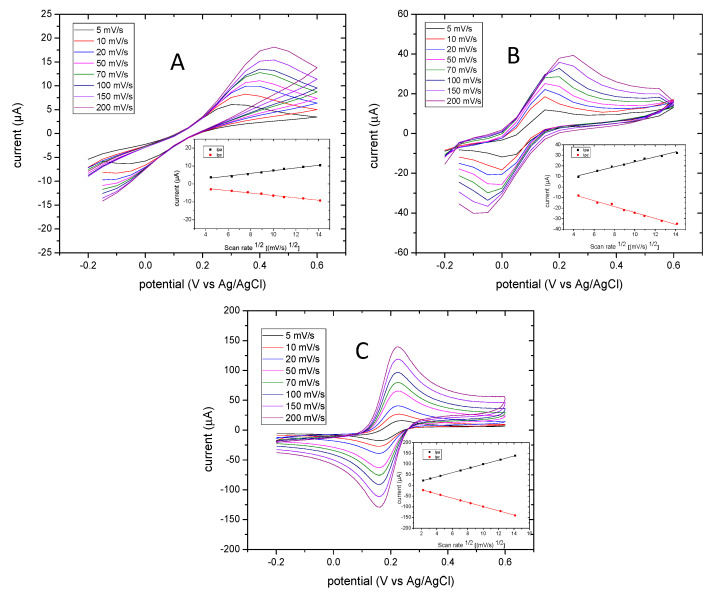
Cyclic voltammograms of 1 mM [Fe(CN)6]^3−^^/4−^ on non-cleaned electrodes (**A**), cleaned electrodes (10 min) (**B**), and cleaned electrodes (20 min) (**C**). The inset demonstrates the curves of reduction peaks’ current (ipc) and oxidation peaks’ current (ipa) versus the square root of the scan rate ((mV/S)^1/2^). The cyclic voltammograms were carried out at an initial potential of −0.2 V and final potential of 0.6 V vs. the Ag/AgCl reference electrode.

Cyclic voltammetry was applied where 1 M KCl was used as the electrolyte for the standard reaction redox of ferri/ferrocyanide. Non-cleaned electrodes and cleaned electrodes for 10 min and cleaned electrodes for 20 min were compared. Figure 2 shows the cyclic voltammogram background of electrodes before and after cleaning using Plasmon cleaner. It is evident that the potential window of the cleaned electrodes (Figure 2B,C) is narrow compared to that of non-cleaned electrodes (Figure 2A). In the insert in Figure 2, the linear relationship observed between the peak current and the square root of the scan rate with a high linear regression R^2^ of 0.98 is outlined. The anodic (ipa) and cathodic (ipc) current peaks display a sensitive performance in electrodes cleaned for 20 min compared to the other electrodes. The electrochemical performance of cleaned electrodes for 20 min in plasmon cleaner was more stable than others. The other electrodes displayed shifts in the potential as the scan rate increased. It appears that the faradic current was affected by any background current. Table 2 summarizes the influence of the scan rate on the half peak potential (Emid vs. Ag/AgCl) and peak-to-peak separation (∆Ep) of anodic and cathodic peaks. The anodic and cathodic peak ratio was 0.98–1.03 as the scan rate increased performance in electrodes cleaned for 20 min. This showed the reversible reaction of [Fe(CN)_6_]^3−/4−^.

The potential of the oxidation peak as the scan rate increased for electrodes cleaned for 20 min was 0.19–0.19 V, and that of electrodes cleaned for 10 min was 0.2–0.3 V. Electrodes that had not been cleaned had a potential oxidation peak of 0.3–0.45 V as the scan rate increased. This indicates that the cleaning process can positively enhance electron transfer, and that a 20-min cleaning time is sufficient to overcome the limitations of diffusion processes. Moreover, the value of ∆Ep at 200 mV/S presented by non-cleaned electrodes was too high (300 mV), which indicates that some sort of contamination occurred on the electrode surface. Cleaning the surface electrodes was important as, after 10 min and 20 min, decrements of the value ∆Ep were observed, which were 163 mV and 58 mV, respectively. Considering the theoretical value of ∆Ep is 59 mV for single electron transfer of [Fe(CN)_6_]^3−/4−^, the closest experimental value achieved was 58 mV. Therefore, treatment with oxygen plasma for 20 min was constantly used for further electrochemical investigations.

### 3.2. Optimization of Electrochemical Measurement

**Figure 3:**
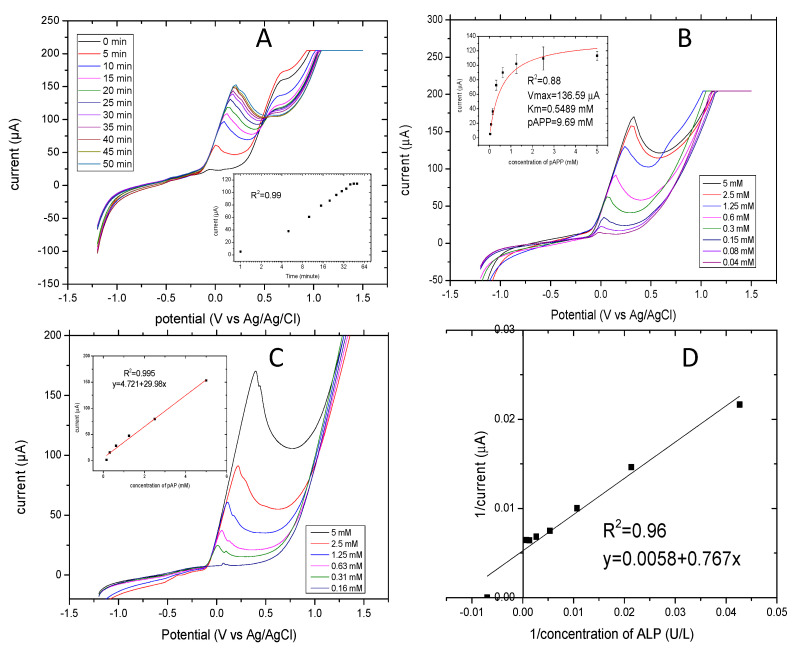
Linear sweep voltammograms of electrochemical optimization of ALP release applied at a potential of −1.2 V to −1.5 V, and a scan rate of 100 mV/S. (**A**) The linear sweep voltammograms of a concentration of pAP of 0.16–5 mM and in the insert is the standard curve of the current response. (**B**) Optimization of the reaction time for the enzymatic assay of ALP release and in the inset is current response versus time. (**C**) Optimization of pAPP concentrations 0.2–5 mM pAPP and in the insert is the current response versus concentrations of pAPP. (**D**) The calibration curve of ALP activity ranging from 1.5–1500 U/L fitted by the Lineweaver-Burk model.

For the electrochemical detection of mammalian ALP release, the electroactive compound p-aminophenol (pAP) is produced from the enzyme reacting with a p-aminophenyl phosphate substrate. The linear sweep voltammetry detection technique was applied to read the current generated at different concentrations of the product pAP. Figure 3A shows the linear sweep voltammograms of different concentrations of pAP (0.16–5 mM). The measurements were carried out at a scan rate of 100 mV/S and at a range of potentials between −1.2 and 1.5 V. The current response of the pAP increased in potentials at points between 0 and 0.3 V. The insert curve shows the standard curve of the mass-transfer limited current of pAP. Furthermore, the values of intercept and slope of regression lines from these six concentrations were calculated as 5.931 and 11.866. The current response for the oxidation of pAP indicated a good linear relationship, where R^2^ was 0.996. pAP (5 mM) generated 180 μA, and the lowest concentration of 0.16 mM generated 20 μA. These results indicate that the voltammetric detection of pAP did not affect the electrode surface during the assay. 

The electrochemical determination of ALP release versus time was performed and showed a linear dependence. When time of incubation increased, the ALP release increased. Figure 3B shows the linear sweep voltammograms of current increased as incubation time increased, reaching a steady state at 40 min. The linear sweep voltammograms were applied at 100 mV/S and at potentials ranging from −1.2 to 1.5 V vs. Ag/AgCl. In the insert of Figure 3B, the values of current increased rapidly up to 10 min and then continued slowly up to 40 min. They levelled off at between 40 and 50 min. The oxidation peak started to increase with increasing time after 10 min, so 10 min was taken as the optimum incubation time for subsequent measurements. This corresponds to data already published by Sappia et al. [43]. The linearity relationship had a very good coefficient regression R^2^ of 0.998.

The optimal concentration of substrate for the evaluation of enzyme activity using this method was investigated. The current responses of pAPP substrate at different concentrations ranging from 0.1 to 5 mM are shown in Figure 3C. ALP release was determined from the constant cell number of 250 × 10^3^ cells/mL. The cells were incubated with the substrate concentration for 10 min at 37 °C. The current responses reached a plateau at 1.25 mM of pAPP. The current values were fitted using the Michaelis-Menten model with a coefficient regression R^2^ of 0.88. The corresponding Imax and km were 136.59 µA and 0.548 mM. The corresponding concentration of pAPP at 95% of Imax was 9.69 mM. The optimal concentration of pAPP was almost 20 times higher than the km [44].

Linear sweep voltammetry was applied for different concentrations of ALP (1.5–1500 U/L) to allow for conversion of the unit and to compare it to the absorbance values. Figure 3D shows the Lineweaver–Burk plots of the current responses (1/current) versus ALP concentration (1/concentration) as obtained by linear sweep voltammetry with good linear regression of R^2^ of 0.99. The intercept (Imax) and the slope (km/Imax) were 0.0058 and 0.767, and the LOD was 0.043 U/L.

### 3.3. Linearity Performance of ALP Release vs. Cell Number

**Figure 4:**
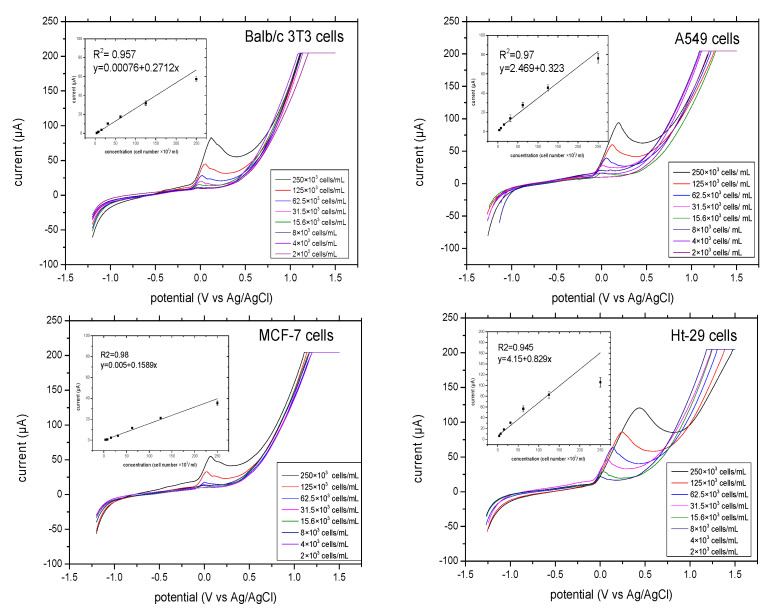
Linear sweep voltammograms of ALP release for each cell range from 2–250 × 10^3^ cells/mL at a scan rate of 100 mV/S, incubation time of 10 min and potential range from −1.2–1.5 V. In the insert, the linearity performance of the ALP release from the given cells versus the current responses with linear regression analysis is outlined. All the measurements were applied in triplicate in separate graphite-SPE in the presence of 9.7 mM pAPP and at final volume of 70 μL.

The linear relationship between ALP release and cell number was analysed. The final concentration of substrate pAPP was about 10–20-fold higher than the km. The linear voltammograms of ALP release from different concentrations of cells (4, 8, 16, 31, 63, 125, and 250 ×10^3^ cells/mL) were determined for each cell line. Figure 4 illustrates the linear sweep voltammetry that was performed at a scan rate of 100 mV/S and at potentials that ranged from −1.2 V to 1.5 V. The potential of the anodic response shifted as the cell number increased. The results are as follows: ALP release from Balb/c 3T3 cells shows oxidation of pAP beginning at −100 mV and ending at 150 mV. ALP release from A549 cells and MCF-7 cells shows oxidation of pAP beginning at −100 mV and ending at 200 mV. Ht-29 cells had slightly wider potential. We found oxidation of pAP beginning at −100 mV and ending at 300 mV. The oxidation peaks displayed by ALP release of the three cells, Balb/c 3T3, A549 and MCF-7, were narrower than those of Ht-29 cells.

There was good linear correlation between the current response and ALP released from the four cells. The cells of Balb/c 3T3 showed the highest value of ALP released at 75 μA, while MCF-7 cells had 50 μA, which was also the lowest value of ALP released among other cells at a concentration of 250 × 10^3^ cells/mL. At the lowest concentration of 4 × 10^3^ cells/mL, the MCF-7 cells showed a current response of 10 μA, while that of Balb/c 3T3 cells was 9 μA. Both showed a good linear response R^2^ of 0.95 and 0.98 for Balb/c 3T3 cells and MCF-7 cells. ALP release from A549 cells and Ht-29 cells showed higher values of currents at 80 μA and at 100 μA, respectively, and also a good linear response (R^2^ = 0.94 for A549 cells and 0.94 for Ht-29 cells). Moreover, the current response in Ht-29 cells had levelled off from the concentration of 1.25 × 10^3^ cells/mL, which indicates that the substrate concentration was running out. Ht-29 cells can release higher amounts of ALP compared to other cells. Therefore, this showed the importance of optimizing substrate concentration for each cell type.

### 3.4. Concentration of the Substrate pAPP from Adhesion Cells

**Figure 5:**
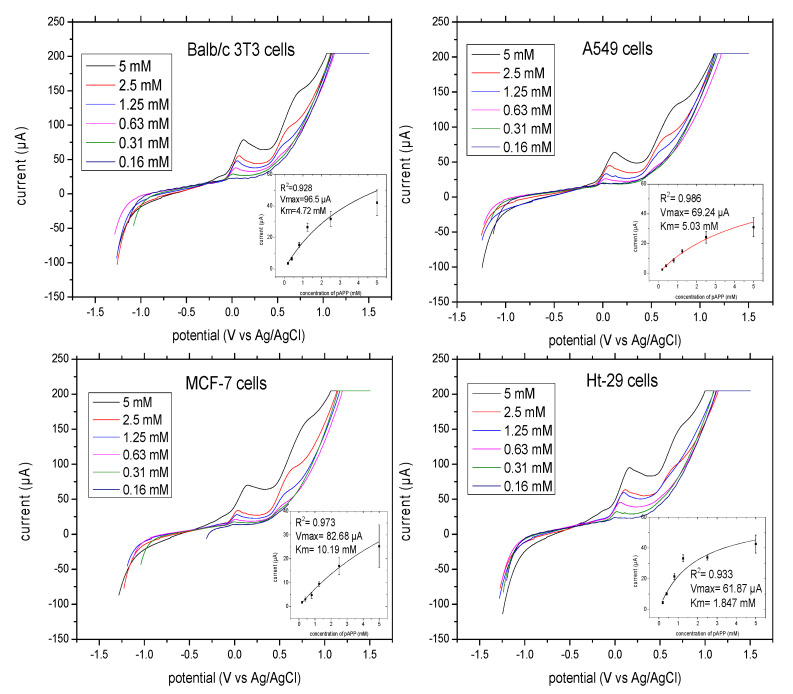
Linear sweep voltammograms of ALP release for adhesion cells in the presence of different concentrations of substrate pAPP (2–5 mM) at a scan rate of 100 mV/S, potential range of −1.2–1.5 V and incubation time of 10 min. In the insert, the concentration of pAPP substrate versus the current responses with non-linear regression analysis is outlined. All the measurements were applied in triplicate in separate graphite-SPE and at final volume of 70 μL.

The optimal concentration of pAPP for the evaluation of ALP release from adhesion cells was investigated. The linear sweep voltammograms of the pAP formed by ALP release from Balb/c 3T3 cells, A549 cells, MCF-7 cells, and Ht-29 cells with different concentrations of substrate ranging from 0.2 to 5 mM pAPP are illustrated in Figure 5. The measurements were taken at a scan rate of 100 mV/S, initial potential of −1.2 V and final potential of 1.5 V. pAP formed by enzymatic reaction at 37 °C after 10 min was measured. The oxidation peaks of pAP began at 0.0 mV and ended at 150 mV for all cells. The current values were plotted against substrate concentration and fitted by the Michaelis–Menten model (Figure 5, insert). The enzymatic reaction of ALP release from Balb/c 3T3, A549, MCF-7, and Ht-29 cells versus different concentrations of the substrate pAPP displayed good non-linear regression, with R^2^ = 0.97. The km values of pAPP from each cell line were as follows: Balb/c 3T3 = 4.75 mM, A549 = 5.03 mM, MCF-7 = 10.19 mM and Ht-29 = 1.85 mM. The Imax values were as follows: Balb/c 3T3 = 96.5 μA, A549 = 69.24 μA, MCF-7 = 82.68 μA and Ht-29 = 61.67 μA. The corresponding concentrations of pAPP at 95% of Imax were as follows: 47.5 mM for Balb/c 3T3 cells; 50.3 mM for A549; 101.9 mM for MCF-7; and 18.5 mM for Ht-29. The differentiation of the parameters indicates the different activity of ALP in each cell line, where the colon cancer cell lines exhibited the highest activity and the breast cancer cell lines had the lowest activity according to their substrate concentration consumed at half maximal velocity.

### 3.5. Comparative Study of ALP Activity

**Figure 6:**
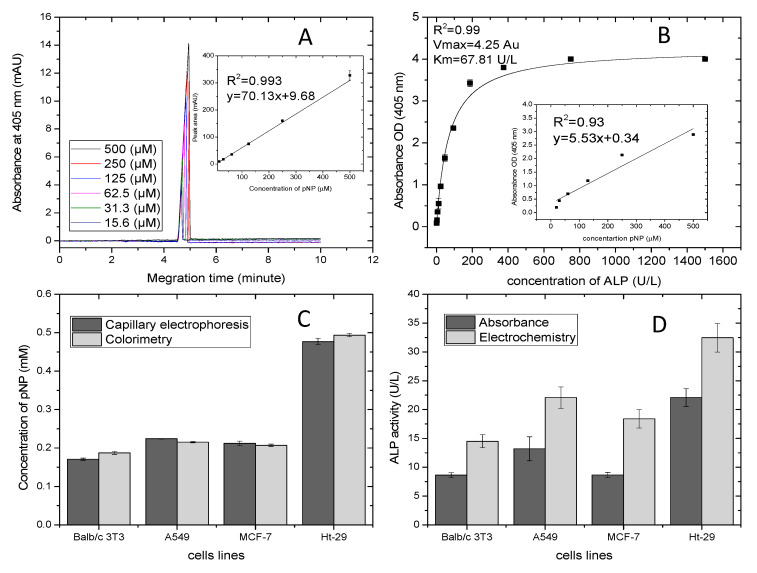
Comparative studies of absorbance and electrochemistry toward ALP release from living cells. (**A**) Electropherograms of pNP concentration and in the insert is the linear trend of peak areas. (**B**) Calibration curve of absorbance values relating to ALP concentration ranging from 1.5–1500 U/L, fitted by the Michaelis-Menten model, and in the insert is the linear trend of absorbance to pNP concentration (15–500 μM). (**C**) Histograms of ALP release measured by capillary electrophoresis and colorimetry. (**D**) Histograms of ALP release measured by absorbance and electrochemistry.

An electrochemical optimized assay was compared to absorbance outputs using the optical active substrate pNPP and two different methods: capillary electrophoresis and colorimetry. The former was applied due to its selective monitoring of p-nitrophenol and because it eliminates any interference with endogenous ALP in the sample. The latter is a standard method for ALP analysis. The analysis was carried out in the presence of 70 μL of the product pNP in the range of 15–500 μM and 30 μL of sample mixed with of 6 mM of pNPP. Figure 6A shows electropherograms of different concentrations of p-nitrophenol in the presence of 1 mM DEA adjusted to pH = 9.5, temperature 20 °C, voltage of 15 kV and wavelength of 405 nm. The measurements of pNP taken in a fused-silica capillary with an effective length of 15 cm and a diameter of 50 μm displayed peaks at migration time of 5 min. This also was confirmed by other studies [45,46,47]. The separation peaks were affected by the length of incubation time and by applied voltage [45]. CE is an ideal assay for further investigation of ALP isoenzymes, as it performs sensitive detection in alkaline buffer. This is an appropriate environment for ALP. The insert details the calibration plot of pNP concentrations versus the peak areas, which displayed very good linear regression of R^2^ = 0.99, slope of 70.13 mAU/µM and intercept of 9.68 mAU. This was used for normalizing the peaks’ areas of ALP release from living cells with values obtained by colorimetric analysis. Figure 6B shows the calibration curve of absorbance versus various concentrations of ALP ranging from 1.5–1500 U/L and fitted by the Michaelis–Menten model. The standards had 30 μL of sample dissolved with pNPP 6 mM in buffer DEA and were incubated for 30 min. Data in triplicate were plotted using origin software. The correlation coefficient was found to be 0.99, and the Vmax and km of the curve were found to be 4.25 and 67.81 U/L. The correlation slope (km/Vmax) and intercept (1/Vmax) were calculated from the constants and were used for comparing data with the electrochemical assay. The insert outlines the linearity studies of colorimetric assay carried out in the presence of different concentrations of pNP (14–500 µM). The regression analysis was 0.99, and the slope and intercept of the curves were found to be 5.53 and 0.34. These were used for normalizing data and comparing them with the capillary electrophoresis assay. Figure 6C shows the normalizing data of ALP release from cells represented by formation of the product pNP. Capillary electrophoresis and colorimetric assay displayed good correlation. No significant difference (*p* > 0.05) resulted from the two methods. It is obvious that the lowest ALP level was determined by Balb/c 3T3 cells, which was 0.16–0.17 mM, whereas the highest level of ALP was of colon cancer cells and was in the range of 0.48–0.50 mM. Lung and breast cancer cells had almost the same levels of ALP (0.21–0.22 mM and 0.20–0.21 mM, respectively). Moreover, the ALP of lung cancer was slightly higher than breast cancer, and results showed very small standard deviation. Figure 6D outlines details of the comparative analysis performed on the absorbance and optimized electrochemical assay of ALP release from living cells. The electrochemical or optical data of ALP release from cells were normalized using the Lineweaver–Burke equation shown in Figure 3C and the Michaelis-Menten model shown in Figure 6B. There was a significant difference (*p* > 0.05) in the activity of ALP release from cells between electrochemical and optical assays. The electrochemical results of ALP activity from Balb/c 3T3, A549, MCF-7 and Ht-29 were higher than ALP activity obtained by absorbance analysis. This optimization demonstrated that evaluation of ALP release by electrochemical assay was more sensitive than by optical assay. Moreover, it showed faster detection as the optical active substrate needed 30 min for evaluating ALP release in samples, while that of the electro active substrate only needed 10 min.

## 4. Conclusions

Alkaline phosphatase is a cancer biomarker, and the monitoring of ALP release from cells contributes to an understanding of the basis of diseases and the progression of cancer. The aim of this paper was to develop a methodology for monitoring ALP release from two types of cells: embryo fibroblast cells (Balb/c 3T3) and cancer epithelial cells (A549, MCF-7, Ht-29), using electrochemical analysis. At alkaline pH, the enzyme ALP hydrolyzes the non-electroactive p-aminophenol phosphate to generate the electroactive, p-aminophenol. The electrochemical behaviour of the sensors used was investigated using cyclic voltammetry and using the solution [Fe(CN)_6_]^3−/4−^ as the model for single transfer electrons. A cleaning duration of 20 min for the sensors in the Plasmon cleaner gives good electrochemical behaviour. The anodic and cathodic peak ratio was almost 1 (ia/ic = 1), and the half peak potential (Emid vs. Ag/AgCl) showed a reversibility reaction. The peak-to-peak separation (∆Ep) of anodic and cathodic peaks was 62 mV. Linear sweep voltammetry detection techniques that have a wide window of potential were applied using graphite-SPE at a scan rate of 100 mV/S and at a potential ranging from −1.2 V to 1.5 V vs. a reference electrode of Ag/AgCl. This demonstrated the standard curve of pAP, and allowed for observation of the potential where oxidation peaks occur. This was at 0–0.15 V of the current response (0–150 µA) displayed when the concentration of pAP (0.16–5 mM) was increasing. The oxidation peaks of pAP began at −100 mV and ended at 300 mV as the concentration increased. The samples were incubated with substrate for different amounts of time. They displayed a positive linear dependence and 10 min was selected as the time to do the rest of the experiments. The substrate pAPP was optimized using detached cells and had a Michaelis constant (km) of 0.548 mM, (Imax) of 136.59 and an optimal p-APP concentration of 9.69 mM. A calibration plot was obtained based on the optimization measurements from a range of 1–1500 U/L. ALP release was determined from various cell numbers for linearity analysis. All cells exhibited linear trends with good regression analysis. The pAPP substrate was investigated during cellular adhesion, and km and Imax were calculated for each cell line. Capillary electrophoresis and colorimetric methods were applied for comparative analysis. CE allows for the detection of ALP release with substrates at the same time, which can be used for distinguishing ALP iso-enzymes. The samples were introduced onto the capillary automatically. The average detection time for p-nitrophenol was 5 min. Colorimetry, which is the standard assay of ALP in clinical analysis, showed compatible values with CE. A calibration curve of ALP was created to allow for comparison of the data obtained by optical and electrochemical analysis. This revealed a significant difference between the two, indicating that the electrochemical investigation resulted in a more sensitive and rapid assay than the optical analysis.

## Figures and Tables

**Table 1:** A summary of the cell number and the composition of media used in this study.

Cell Line	Concentration (×10^3^ Cells/mL)	Media	Supplements
**Balb/c 3t3**	40	DMEM	10% NBCS
**A549**	40	DMEM	10% FBS
**MCF-7**	40	MEME	10% FBS
**Ht-29**	80	McCoy’s 5A	10% FBS

**Table 2:** A summary of the influence of the scan rate on the half peak potential (Emid vs. Ag/AgCl) and peak-to-peak separation (∆Ep) of anodic and cathodic peaks.

Scan Rate	^a^ E_mid_ vs. Ag/AgCl			^b^ ∆Ep (mV)		
	0 min	10 min	20 min	0 min	10 min	20 min
**5**	0.2285	0.0768	0.198	175	146.4	76
**10**	0.2465	0.08145	0.194	207	143.1	68
**20**	0.261	0.09155	0.192	230	144.9	68
**50**	0.2715	0.1163	0.192	247	137.4	64
**70**	0.277	0.1225	0.191	260	151	62
**100**	0.282	0.1305	0.191	270	153	62
**150**	0.294	0.135	0.194	288	162	64
**200**	0.298	0.1585	0.193	300	163	58

^a^ Measured from the value of 1/2(*Epc* + *Epa*) versus Ag/AgCl reference electrode. ^b^ *∆Ep = Epa − Epc*.
